# “Simplified International Recommendations for the Implementation of Patient Blood Management” (SIR4PBM)

**DOI:** 10.1186/s13741-017-0061-8

**Published:** 2017-03-17

**Authors:** Patrick Meybohm, Bernd Froessler, Lawrence T. Goodnough, Andrew A. Klein, Manuel Muñoz, Michael F. Murphy, Toby Richards, Aryeh Shander, Donat R. Spahn, Kai Zacharowski

**Affiliations:** 1 Department of Anaesthesiology, Intensive Care Medicine and Pain Therapy, University Hospital Frankfurt, Theodor-Stern-Kai 7, 60590 Frankfurt am Main, Germany; 2 Department of Anaesthesia, Lyell McEwin Hospital, South Australia, Australia; 3 Pathology and Medicine (Hematology), Stanford University, Stanford, CA USA; 4 Department of Anaesthesia and Intensive Care, Papworth Hospital, Cambridge, UK; 5 Transfusion Medicine, School of Medicine, University of Málaga, Málaga, Spain; 6 NHS Blood and Transplant, Oxford University Hospitals NHS Foundation Trust, University of Oxford, Oxford, UK; 7 Centre for CardioVascular and Interventional Research (CAVIAR), University College London, Rockerfellow Building, University Street, London, UK; 8 Department of Anaesthesiology and Critical Care and Hyperbaric Medicine, Englewood Hospital and Medical Center, TeamHealth Research Institute, Englewood, NJ USA; 9 Institute of Anaesthesiology, University of Zurich and University Hospital of Zurich, Zurich, Switzerland

**Keywords:** Patient blood management, Anemia, Patient outcome

## Abstract

**Background:**

More than 30% of the world’s population are anemic with serious medical and economic consequences. Red blood cell transfusion is the mainstay to correct anemia, but it is also one of the top five overused procedures and carries its own risk and cost burden. Patient blood management (PBM) is a patient-centered and multidisciplinary approach to manage anemia, minimize iatrogenic blood loss, and harness tolerance to anemia in an effort to improve patient outcome. Despite resolution 63.12 of the World Health Organization in 2010 endorsing PBM and current guidelines which include evidence-based recommendations on the use of diagnostic/therapeutic resources to provide better health care, many hospitals have yet to implement PBM in routine clinical practice.

**Method and results:**

A number of experienced clinicians developed the following “Simplified International Recommendations for Patient Blood Management.” We propose a series of simple, cost-effective, best-practice, feasible, and evidence-based measures that will enable any hospital to reduce both anemia prevalence on the day of intervention/surgery and anemia-related unnecessary transfusion in surgical and medical patients, including obstetrics and gynecology.

## Background

Patient blood management (PBM), refers to “the timely application of evidence based medical and surgical concepts designed to maintain hemoglobin concentration, optimize hemostasis and minimize blood loss in an effort to improve patient outcome” (Society for the Advancement of Blood Management (SABM) 2014). Thereby, PBM focuses on preservation of patient’s own blood. It questions the dogma of red blood cell (RBC) transfusion as the primary strategy for the treatment of anemia but also supports the use of appropriate transfusion practice in the hospitalized patient.

PBM should be performed by an institutionally empowered multidisciplinary team considering three most important principles: first, management of the patient’s anemia, which mainly involves early detection of anemia and utilizing nutritional and pharmaceutical treatments to support erythropoiesis, if it is not mainly genetic-related; second, the use of interdisciplinary blood conservation measures to reduce iatrogenic blood loss, including prevention and proactive management of coagulopathy, precise anesthetic and surgical techniques, intra- and postoperative autologous blood conservation techniques, and minimization of phlebotomy volume and frequency; and third, harness tolerance to anemia and patient-centered decision-making to allow optimal blood use involving thorough communication with the patient about the risks and benefits of the various potential interventions (Society for the Advancement of Blood Management (SABM) 2014; Goodnough et al. 2013; Spahn and Goodnough 2013).

Considerable scientific evidence indicates that PBM reduces perioperative blood loss and transfusion needs (Leahy et al. 2017; Gross et al. 2015; Goodnough et al. 2014a; Goodnough et al. 2014b; Moskowitz et al. 2010; Oliver et al. 2014; Theusinger et al. 2014; Leahy et al. 2014; Roubinian et al. 2014; Meybohm et al. 2016a), perioperative morbidity (Leahy et al. 2017; Gross et al. 2015; Moskowitz et al. 2010; Meybohm et al. 2016a), mortality (Leahy et al. 2017; Goodnough et al. 2014a; Moskowitz et al. 2010), length of hospital stay (Goodnough et al. 2014a; Moskowitz et al. 2010), and costs (Leahy et al. 2017; Trentino et al. 2015). In this respect, the WHO has officially been urging member states to implement PBM since 2010 (WHA63.12). There exist also a number of guidelines and standards from professional associations providing detailed evidence-based information and recommendations on PBM in Australia, the USA, and in several European countries (Society for the Advancement of Blood Management (SABM) 2014; National Blood Authority Australia; Joint United Kingdom (UK) Blood Transfusion and Tissue Transplantation Services Professional Advisory Committee 2014; American Society of Anesthesiologists Task Force on Perioperative Blood Management 2015; Government of Western Australia Department of Health; Kozek-Langenecker et al. 2013a; Leal-Noval et al. 2013; Kozek-Langenecker et al. 2013b; Hunt et al. 2015; AABB 2014; NHS Blood and Transplant; AABB 2015; Klein et al. 2016). However, many barriers limit the translation of PBM into clinical practice worldwide (Munoz et al. 2015; Fischer et al. 2015; Vamvakas 2013; Mbanya 2012). These include clear guidance on clinical pathways, lack of knowledge, lack of interdisciplinary commitment, lack of resources, and concerns about risks. In a recent paper (Meybohm et al. 2017), we provided comprehensive bundles of PBM components encompassing more than 100 different PBM measures to facilitate a stepwise implementation process of the most feasible measures.

The aim of this current opinion-based document is to provide clinicians with a working template to start or improve the implementation of PBM practices, specifically through the implementation of simplified recommendations developed with reference to existing best practice guidelines and appropriate key literature on blood transfusion.

## Methods

Experts in PBM were consulted in order to provide consensus on key simple recommendations for the implementation of PBM based on their current practice and experience in Australia, Europe, and the USA. The selection of the authors was intended but covered experts who have been involved in developing a European Guide on Good Practices for Patient Blood Management (P.M., K.Z.), international consensus on the perioperative management of anemia and iron deficiency (P.M., A.A.K., M.M., T.R., A.S., D.R.S.), National Institute for Health and Care Excellence Guidance on blood transfusion (M.F.M.) (Padhi et al. 2015), and landmark papers in the field of PBM (B.F., L.T.G., A.S.). Level of evidence was assigned to each recommendation (level A, multiple randomized clinical trials or meta-analyses; level B, single randomized trial or non-randomized studies; level C, consensus opinion of experts, case studies, or standard-of-care). All authors were asked to vote point-by-point for each recommendation (agreed/not agreed/abstention). If a statement did not achieve 80% or more of the votes, the statement underwent further revision and then entered again into the online voting process. The consensus of recommendation is shown in Table 1.

**Table 1:** Summary of the simplified recommendations with level of evidence (LoE) and consensus of recommendation (CoR)

Recommendations	LoE	CoR (%)
1. Comprehensive project management		
Key leaders; involvement of stakeholders	C	100
Collection of PBM-related metrics	C	90
2. Education program		
Education initiatives; standard operating procedures, clinical protocols, visual aids, checklists; algorithms	C	100
Massive hemorrhage protocols; coagulation and transfusion algorithms	C	100
Online PBM e-learning course	C	100
Education at medical schools and hospital level	C	100
3. Diagnosis and treatment of preoperative anemia		
Preoperative screening, diagnosis, and treatment of anemia	B	100
Intravenous iron if oral iron is not tolerated or if surgery <4–6 weeks	B	100
Erythropoiesis-stimulating agents if nutritional deficiencies have been ruled out, corrected, or both	B	100
Elective surgery should be postponed until preoperative anemia has been classified and treated, if possible	C	100
4. Reduction of iatrogenic diagnostic-/surgery-related blood loss		
Avoiding unnecessary laboratory tests, lower frequency of sampling, using the smallest collection tube size	C	100
Closed in-line flush blood sampling devices for arterial (and central) lines	B	100
Appropriate cessation strategies for anticoagulation and antiplatelet therapy	C	100
Intraoperative approaches (meticulous hemostasis, minimally invasive surgery, laparoscopic surgery, diathermy dissection, physicians’ mindfulness regarding limiting blood loss, topical hemostatic agents)	B	100
Coagulation algorithm (preoperative assessment, ensuring basic conditions for hemostasis, reversal of anticoagulants, point-of-care diagnostics, optimized coagulation management, use of clotting factor concentrates)	B	100
Tranexamic acid	A	100
Autologous blood cell recovery (cell salvage)	A	100
5. Optimal blood component use with patient-centered clinical decision support		
Physician order entry with clinical decision support	B	100
Patient’s informed consent prior transfusion; hand-written/computer-generated forms with detailed outline of transfusion benefits, risks, and alternatives; information in discharge summary; patient’s own preferences	C	80

We would like to stress that this paper contains the authors’ independent opinions based on experience, as well as evidence-based practices supported by clinical studies. No pharmaceutical company has funded the development or writing of the manuscript.

## Simplified International Recommendations for the Implementation of Patient Blood Management (SIR4PBM)

### Comprehensive project management

Each hospital should appoint key leaders for PBM project management (this could be a physician, a nurse, or the institution’s patient safety officer), who should have a central role in charge of communication, education, and documentation. Communication should involve the following stakeholders: chief medical officer, chief executive officer, surgeons, anesthesiologists, intensive care specialists, nurses, transfusion medicine specialists, transfusion committee, gastroenterologists, hematologists, cardiologist, general practitioners, finance administrative and quality management personnel, central laboratory, information technology department, and patients’ representatives (level C).

PBM-related metrics and blood usage should be collected to monitor implementation success and allow identification of potential areas for improvement. PBM-related metrics should include proportion of patients who are anemic and receive treatment, use of blood conservation techniques, and use of hemostatic products and blood components. Data should be audited at ward, specialty, and divisional level with regular feedback to all hospital staff. Further consideration should be given for these metrics to be utilized as a quality improvement marker for appraisal of staff groups (level C).

### Education program

In clinical areas, such as preoperative clinic/preadmission testing units, operating room, and intensive care unit, PBM education initiatives need to be addressed with physicians and nurses. Standard operating procedures, clinical protocols, visual aids, and checklists are crucial. They should include outpatient and preoperative protocols and ward-based transfusion algorithms. Also, training on the use of available massive hemorrhage protocols should be provided (level C). These massive hemorrhage protocols including specific coagulation and transfusion algorithms for postpartum, trauma, transplant, or cardiac surgery should be in place and encourage early detection, definitive intervention, and treatment of acute hemorrhage (Kozek-Langenecker et al. 2013a; Hunt et al. 2015; Kozek-Langenecker 2014; Spahn et al. 2013; Rossaint et al. 2013) (level C). A mandatory online PBM e-learning course may further underpin education initiatives (Meybohm et al. 2016b; National Service Scotland; BloodSafe eLearning Australia) (level C). Ideally, education on PBM should be initiated among undergraduates at medical schools and continued at hospital level (level C).

### Diagnosis and treatment of preoperative anemia

Preoperative screening includes evaluation and management of anemia. From a practical point of view, patients scheduled for surgical procedures with expected blood loss (>500 ml) or a ≥10% probability of RBC transfusion should be identified and assessed at the earliest opportunity and be screened for iron deficiency and other likely causes of anemia (Government of Western Australia Department of Health; AABB 2014; National Service Scotland; Carless et al. 2010; Goodnough and Schrier 2014; Shah et al. 2015; Muñoz et al. 2017) (level B). Serum ferritin level <30 ng/ml, transferrin saturation <20%, and/or microcytic hypochromic red cells (mean corpuscular volume <80 fl; mean corpuscular hemoglobin <27 pg) are indicative of iron deficiency. In the presence of inflammation or transferrin saturation <20%, a ferritin >100 ng/ml points to functional iron deficiency (iron sequestration). The availability of an easy-to-follow, diagnostic algorithm is desirable (Goodnough and Schrier 2014; Muñoz et al. 2017). Intravenous iron is efficacious and safe (Auerbach and Macdougall 2014) and should be used in patients in whom oral iron is not tolerated or if surgery is planned in less than 4–6 weeks after the diagnosis of iron deficiency (Shander et al. 2014a; Froessler et al. 2016; Bisbe et al. 2011; Munoz et al. 2014) (level B). Erythropoiesis-stimulating agents might be suggested for anemic patients in whom nutritional deficiencies have been ruled out, corrected, or both (Goodnough et al. 2011; Voorn et al. 2016; Weltert et al. 2015) (level B). Elective surgery should be postponed until preoperative anemia has been appropriately classified and treated, if possible (level C).

### Reduction of iatrogenic diagnostic-/surgery-related blood loss

Blood loss associated with invasive laboratory testing can either cause or aggravate hospital-acquired anemia which is associated with increased length of stay and complications (Koch et al. 2015). Blood testing should not be “routined” but informed. Reduction of blood drawn for laboratory analyses can be achieved by avoiding unnecessary laboratory tests and lower frequency of sampling (Raad et al. 2016) and using the smallest collection tube size that is practical for the required analysis, which is often pediatric-size bottles (level C). However, there are several difficulties with these bottles (e.g., overfill and underfill of tubes, large IT labels not fitting on small tubes, issues with the bottles not fitting into automated analyzers). Further reduction of phlebotomy-associated blood loss can be achieved by using closed in-line flush blood sampling devices for arterial (and central) lines (Koch et al. 2015; Ranasinghe and Freeman 2014; Fischer et al. 2014; Mukhopadhyay et al. 2010) (level B).

Reduction of surgery-related blood loss starts from the preoperative stage with appropriate cessation strategies for anticoagulation and antiplatelet therapy (level C). Intraoperative approaches such as advanced anesthetic and surgical techniques with meticulous hemostasis including minimally invasive surgery, laparoscopic surgery, judicious use of diathermy dissection, physicians’ mindfulness regarding limiting blood loss, and application of topical hemostatic agents (Shander et al. 2014b; Menkis et al. 2012; Emilia et al. 2011; Anastasiadis et al. 2016) (level B).

Advanced perioperative coagulation monitoring is crucial for avoiding unnecessary blood loss. Adequate coagulation management needs to be a precondition before RBC transfusion is considered. In this respect, the use of a coagulation algorithm is recommended (Weber et al. 2012; Weber et al. 2014), encompassing preoperative assessment (Munoz et al. 2016) and ensuring basic conditions for hemostasis (e.g., temperature, calcium, pH), reversal of anticoagulants, point-of-care diagnostics in bleeding (e.g., coagulopathic) patients (if available), and optimized coagulation management with the use of clotting factor concentrates (Kozek-Langenecker et al. 2013a; Kozek-Langenecker 2014; Meybohm et al. 2013; Weber et al. 2013) (level B). To reduce surgical blood loss, tranexamic acid should be used unless contraindicated (Perel et al. 2013; Ker et al. 2013) (level A).

The use of intraoperative autologous blood collection and re-transfusion (cell salvage) should be standardized including indications and contraindications (Carless et al. 2010). The use of (washed) cell recovery is highly recommended in surgical settings where blood loss is routinely or anticipated over 500 ml as it reduces the rate of exposure to allogeneic RBC, risk of infection, and length of hospital stay (Meybohm et al. 2016c) (level A).

### Optimal blood component use with patient-centered clinical decision support

In order to optimize utilization of allogeneic blood products/components and to identify the ordering physician, it is beneficial to adopt a physician order entry with a clinical decision support based on electronic medical records (Goodnough et al. 2014a; Oliver et al. 2014) (level B). Thereby, indication for transfusion considering patient-specific factors (e.g., age, diagnosis, co-morbidity, surgical or non-surgical setting), signs/symptoms of acute anemia, laboratory values (e.g., hemoglobin), presence or absence of bleeding, and physiologic factors (e.g., oxygenation, hemodynamic status) can be confirmed with required checkboxes (Goodnough et al. 2016).

Patient’s informed consent should be obtained prior transfusion of allogeneic blood products (components). In emergency cases where it is not possible to obtain consent, patient should be informed as soon as possible after transfusion. A hand-written or computer-generated forms (ideally a separate consent form) should be used that is comprehensive and includes a detailed outline of transfusion benefits, risks, and alternatives (ISBT ethic code for blood donation and transfusion (International Society of Blood Transfusion 2000)). It is necessary to effectively communicate the risks and benefits of the various potential interventions and to decide on the right course of action together with the patient. It may further be recommended that any transfusion of allogeneic blood products (components) should be mentioned in the discharge summary. The patient’s own preferences and values should be considered when developing a medical plan (level C).

## Conclusions

To enable any hospital to reduce both anemia prevalence and anemia-related unnecessary blood transfusion, here, we provide simple, cost-effective, best-practice, feasible, and evidence-based measure recommendations developed with reference to existing best practice guidelines. As the level of evidence is still low for some recommendations, we suggest that further studies are needed to elucidate the potential role of these PBM measures.

## Abbreviations

PBM: Patient blood management
RBC: Red blood cells
